# Targeted massive parallel sequencing: the effective detection of novel causative mutations associated with hearing loss in small families

**DOI:** 10.1186/1750-1172-7-60

**Published:** 2012-09-03

**Authors:** Jeong-In Baek, Se-Kyung Oh, Dong-Bin Kim, Soo-Young Choi, Un-Kyung Kim, Kyu-Yup Lee, Sang-Heun Lee

**Affiliations:** 1 Department of Biology, College of Natural Sciences, Kyungpook National University, Daegu, 702-701, South Korea; 2 Department of Otolaryngology, College of Medicine, Kyungpook National University, Daegu, 700-721, South Korea

**Keywords:** Hearing loss, Heterogeneous, Next-generation sequencing, Mutation, Gene

## Abstract

**Background:**

Hereditary hearing loss is one of the most common heterogeneous disorders, and genetic variants that can cause hearing loss have been identified in over sixty genes. Most of these hearing loss genes have been detected using classical genetic methods, typically starting with linkage analysis in large families with hereditary hearing loss. However, these classical strategies are not well suited for mutation analysis in smaller families who have insufficient genetic information.

**Methods:**

Eighty known hearing loss genes were selected and simultaneously sequenced by targeted next-generation sequencing (NGS) in 8 Korean families with autosomal dominant non-syndromic sensorineural hearing loss.

**Results:**

Five mutations in known hearing loss genes, including 1 nonsense and 4 missense mutations, were identified in 5 different genes (*ACTG1, MYO1F, DIAPH1, POU4F3* and *EYA4*), and the genotypes for these mutations were consistent with the autosomal dominant inheritance pattern of hearing loss in each family. No mutational hot-spots were revealed in these Korean families.

**Conclusion:**

Targeted NGS allowed for the detection of pathogenic mutations in affected individuals who were not candidates for classical genetic studies. This report is the first documenting the effective use of an NGS technique to detect pathogenic mutations that underlie hearing loss in an East Asian population. Using this NGS technique to establish a database of common mutations in Korean patients with hearing loss and further data accumulation will contribute to the early diagnosis and fundamental therapies for hereditary hearing loss.

## Background

A number of hereditary disorders that follow a Mendelian inheritance pattern are genetically heterogeneous. Hereditary hearing loss is one such heterogeneous disorder, and it may be caused by a multitude of genes. Currently, mutations in 63 genes have been found to be associated with hearing loss. However, there are 54 candidate chromosomal loci at which causative genes have not yet been identified, although classical genetic studies such as linkage analysis have predicted that these loci contain novel hearing loss-associated genes (Hereditary Hearing loss Homepage, http://hereditaryhearingloss.org). Due to the limitation of the Sanger sequencing method, which is highly expensive and time-consuming, it has been difficult to sequence the hundreds of genes in these candidate chromosomal loci. Therefore, it has been nearly impossible to identify the precise pathogenic mutations in affected individuals from small families who cannot be examined either through linkage analysis or standard capillary sequencing analysis. Next-generation sequencing (NGS) can overcome these limitations through its ability to perform parallel sequencing of billions of nucleotides at a low cost and high speed [1-4]. The capacity to simultaneously screen thousands of target genes makes this technique an especially powerful tool for detecting pathogenic mutations that cause heterogeneous disorders such as hereditary hearing loss. In 2010, Walsh et al. performed whole-exome sequencing in a Palestinian family with hereditary hearing loss and identified a novel mutation in the gene, *GPSM2*, which had been hidden in the autosomal recessive hearing loss locus (DFNB32) first identified by Masmoudi et al. in 2003 [5]. However, there have been no reports of the detection of pathogenic mutations by screening candidate hearing loss genes in small families without using linkage analysis.

Therefore, based on the hypothesis that causative mutations of hearing loss are more likely to exist in known hearing loss associated-genes than in novel genes, we performed exon capture and resequencing using Illumina library generation and Solexa sequencing methods.

## Subjects and methods

### Families and clinical evaluation

**Figure 1:**
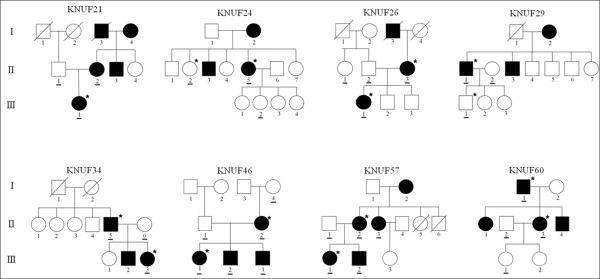
**Pedigree diagrams of 8 Korean families with autosomal dominant hearing loss.** An asterisk in the upper right corner of the symbol indicates an individual whose genomic DNA was analyzed by targeted sequencing. Co-segregation of the candidate variations detected in the targeted sequencing and the phenotype of hearing loss was confirmed via Sanger sequencing of DNA from the individuals marked by a bar. Squares and circles represent males and females, respectively. Filled symbols represent affected individuals, and slashes indicate deceased individuals.

Eight small Korean families with hereditary hearing loss were recruited from the Department of Otorhinolaryngology-Head and Neck Surgery at the Kyungpook National University Hospital in Daegu, South Korea (Figure 1). A total of 31 individuals, including 18 affected and 13 unaffected members, participated in this study. A clinical questionnaire excluded any history of other diseases and environmental factors, including infection, ototoxicity and noise. Physical examinations ruled out the probability of syndromic hearing loss. The hearing level in all of the participants was evaluated by audiological tests, including pure-tone audiometry (PTA) and auditory brainstem response (ABR). PTA was calculated as an average of the threshold measured at 0.5, 1.0, 2.0 and 4.0 KHz [6], and air-conduction threshold measurements were performed at 125-8000 Hz. The level of hearing loss is described as follows depending on PTA: normal hearing, below 20 dB; mild hearing impairment, 21 to 40 dB; moderate hearing impairment, 41 to 70 dB; severe hearing impairment, 71 to 95 dB; and profound hearing impairment, above 95 dB. In addition, bone conduction thresholds were measured at 250-4000 Hz to check conductive hearing loss in affected individuals. One hundred unrelated Koreans with normal hearing were recruited from Kyungpook National University Hospital as normal controls. All of the participants provided written informed consent before the study according to the protocol approved by the Ethics Committee of Kyungpook National University Hospital. Genomic DNA from 31 individuals, including 18 patients from 8 families and 100 normal controls, was extracted from peripheral blood or buccal cells using either the FlexiGene DNA extraction kit (QIAGEN, Hilden, Germany) or the Puregene Buccal Cell Core kit, respectively (QIAGEN, Hilden, Germany). All of the subjects were examined for the *GJB2* and the *SLC26A4* gene by Sanger sequencing and were negative for both of the genes.

### Targeted resequencing and variation analysis

Using previously published literature, we selected 80 genes that are associated with sensorineural hearing loss as the target genes for sequencing (Additional file 1: Table S1). Five microgram of genomic DNA was fragmented to approximately 250 base pairs, which was followed by end-repair, adenylation and adapter ligation for library generation using an Illumina sample preparation kit (Illumina, Inc., San Diego, CA, USA). All protein-coding regions and exon-intron boundaries of the target genes were captured by hybridization with designed nucleotide probes. The captured target DNA fragments were sequenced using the Illumina HiSeq 2000 paired-end read sequencing system for 90 cycles per fragment.

All bioinformatics processing and data analysis, including genome alignment, variation detection, filtering and visualization, were performed on the DNAnexus platform (DNAnexus, Inc., Mountain View, USA, http://www.dnanexus.com). The millions of reads that were derived from the targeted sequencing were aligned to the human reference genome hg19 (University of California, Santa Cruz). dbSNP Build 134 was used as a reference for recorded SNPs. The 1000 genome database ( http://www.1000genomes.org) and the Washington University exome database ( http://evs.gs.wathington.edu) were used as references to investigate the novelty and probable pathogenicity of the allelic variations detected in our sequencing approach. The reliability of variations was estimated by the allele frequency and Ref score, which represents the PHRED-encoded probability (quality score) [7,8]. For a probability *p*, the PHRED-encoded quality score *Q* is given by the formula *Q* = -*10log*_10_*p*. Variations with a quality score of less than 20 were regarded as sequencing errors and discarded, and only alleles that appeared in more than 20% of heterozygotes from 8 DNFA families were recognized as potentially real allelic variations. To analyze the possible functional pathogenic effects of the variants, 2 types of prediction programs, SIFT ( http://sift.jcvi.org) and Mutation taster ( http://www.mutationtaster.org), were used.

### Sanger sequencing

Specific exons containing candidate variations were amplified by polymerase chain reaction (PCR) using primer sets designed via Primer3 software ( http://www-genome.wi.mit.edu/cgi-bin/primer/primer3_www.cgi). Each variation was sequenced by the Sanger sequencing method using an ABI PRISM Big Dye Terminator Cycle Sequencing Kit v3.1 and an ABI 3130xl DNA sequencer (Applied Biosystems Corps., Foster City, CA, USA). Finally, the accurate genotype of the variations was confirmed by sequence analysis with the Sequencing Analysis software v5.2 (Applied Biosystems Corps., Foster City, CA, USA) in both the family members and 100 unrelated normal controls. To investigate the significance of the variations, the conservation level of amino acids between other species was estimated using a PhyloP score obtained from the UCSC genome browser ( http://genome.ucsc.deu).

## Results

### Clinical features of the 8 Korean families

All 8 families exhibited a typical autosomal dominant inheritance pattern of hearing loss (Figure 1). Audiological assessments of the affected individuals revealed symmetrical, bilateral, progressive, sensorineural hearing loss that affected both genders (Additional file 2: Figure S1). Patient II-3 of KNUF60 showed asymmetric features (Table 1). According to PTA tests, the severity of hearing loss varied among affected individuals, ranging from mild to profound. The audiogram patterns from all of the patients were distinct, even between members of the same family. None of the affected individuals displayed symptoms of tinnitus, vestibular dysfunction or other clinical abnormalities indicating syndromic hearing loss.

**Table 1:** Clinical features of patients in 8 Korean families carrying autosomal dominant hearing loss

**Family no.**	**Individual no.**	**Gender**	**Age (year)**	**Mean hearing threshold (dB)**		**Shape of audiogram**	**Severity**
				**Left**	**Right**		
KNUF21	III-1^*^	Female	1	90	80	-	Severe
KNUF24	II-5	Female	44	39	45	Reverse U-shaped	Moderate
KNUF26	III-1	Female	18	64	65	Sloping	Moderate-severe
KNUF29	II-1	Male	63	54	54	Sloping	Moderate
KNUF34	II-5	Male	66	56	53	Sloping	Moderate
	III-3	Female	21	54	49	Flat	Moderate
KNUF46	II-2	Female	35	99	98	Sloping	Profound
	III-1	Female	9	43	38	Ascending	Mild
	III-2	Male	7	53	53	Ascending	Moderate
	III-3	Male	5	63	68	Ascending	Moderate-severe
KNUF57	III-1	Female	28	84	89	Ski sloping	Severe
	III-2	Male	27	54	45	Flat	Moderate
KNUF60	II-3	Female	33	91	65	Flat	Severe

* The hearing test for III-1 (KNUF21) was performed by auditory brainstem response (ABR).

### Targeted resequencing and variation analysis

One (KNUF21) or two members per family were selected for targeted sequencing (Figure 1). For these 15 individuals, including 13 patients and 2 normal individuals, we screened 80 candidate deafness genes, including 46 reported hearing loss genes (Additional file 1: Table S1). Target enrichment and massively parallel sequencing using the Illumina HiSeq 2000 paired-end sequencing system produced hundreds of thousands of target mapped reads (tens of millions of bases) with an average length of 90 bases. This sequencing covered more than 90% of the target regions with a mean depth ranging from 43× to 337×. More than 78% of the target regions were covered by 10 or more reads, demonstrating the high quality of the sequencing (Table 2).

**Table 2:** Run statistics and target coverage of NGS in each individual

**Family no.**	**Individual no.**	**On-target sequenced bases (bp)**	**Covered target region (bp)**	**Target coverage (%)**	**Mean depth (**×**)**	**Depth ≥ 10**× **(%)**
KNUF21	III -1	11,555,406	193,337	90.05	54	82.22
KNUF24	II -2	54,927,039	201,347	93.78	256	92.21
	II-5	16,525,672	196,024	91.30	77	85.82
KNUF26	II-3	67,166,957	202,456	94.30	313	92.41
	III-1	9,251,859	192,854	89.83	43	78.26
KNUF29	II-1	12,384,809	195,104	90.87	58	83.71
	III-1	44,392,335	201,109	93.67	207	92.14
KNUF34	II-5	57,045,380	201,906	94.04	263	92.39
	III-3	10,551,804	194,536	90.61	50	82.51
KNUF46	II-2	72,336,591	201,693	93.94	337	92.55
	III-1	14,635,372	195,693	91.15	68	83.51
KNUF57	II-2	51,755,311	200,756	93.51	241	92.20
	III-1	12,911,952	194,467	90.58	60	84.60
KNUF60	I-1	59,181,391	202,284	94.22	276	92.47
	II-3	10,591,967	194,615	90.65	49	82.61

After heterozygous variations were sorted according to the inheritance patterns of each family, quality control filtering was performed based on the PHRED score ( > 20) and variant allele frequency (higher than 20%). This process left hundreds of variations, ranging from 285 to 701 variations per individual (Additional file 3: Table S2). To identify the most probable pathogenic mutations, we applied a prioritization scheme, as described in previous studies [9-11]. First, only heterozygous variants were selected as candidates based on the autosomal dominant inheritance pattern of hearing loss in the families. Next, intergenic or intronic variants were discarded, and only non-synonymous variants in coding sequences and splice-site variants were sorted out as probable pathogenic variants, based on their direct association with the protein expression or function. Because a number of pathogenic mutations have been registered as dbSNPs in international biological databases, all previously reported coding SNPs were ruled out by confirming their allele frequency in several populations and non-pathogenic properties in the NCBI database (National Center for Biotechnology Information, http://www.ncbi.nlm.nih.gov) and HAPMAP project ( http://www.hapmap.org). This approach yielded an average of 12 novel non-synonymous variations located in coding regions in each individual (Additional file 3: Table S2). Most of these novel non-synonymous variations were missense or nonsense mutations caused by single nucleotide substitutions, and a few variations were frame-shift insertions or deletions of a single nucleotide. These variations were checked for co-segregation with hearing loss in the two family members who had been subjected to targeted sequencing, and 10 variations satisfied this criterion.

### Identification of candidate mutations of hearing loss in each family

Variant filtering was performed to identify the most probable causative variants, and 10 non-synonymous variants were found to be significant candidates in 7 families, with the exception of the KNUF26 family. A second co-segregation analysis was performed using the Sanger sequencing method in 14 additional individuals (underlined in Figure 1) from the 7 families, and 3 variations that were not co-inherited with hearing loss were subsequently ruled out from the candidate mutations. Ultimately, 7 variations were confirmed for their co-segregation with hearing loss in these families (Table 3). Although all of these variations were found in genes previously known to be associated with hearing loss, 5 of the 7 variations were novel missense (p.D187H in *ACTG1*, p.P678S in *DIAPH1*, p.E232K in *POU4F3* and p.P1422L in *COL11A2)* or nonsense mutations (p.S288X in *EYA4*) that had not been recorded in any public databases [12-18]. Sanger sequencing confirmed that all 7 variations in the 7 DFNA families were heterozygous in the patients with hearing loss, which was consistent with the inheritance pattern of the disorder.

**Table 3:** Overview of 7 variants showing co-segregation with hearing loss in the families

**Family no.**	**Gene**	**Exon**	**Variation type**	**Nucleotide change**	**Amino acid change**	**PhyloP score**	**Mutation taster**^**†**^	**SIFT**^**‡**^	**Allele frequency in controls**	**References**
KNUF21	*ACTG1*	4	Missense	c.559G>C	p.D187H	2.05	DC	Affected / 0.04	0.00	Novel
KNUF24	*EYA4*	11	Nonsense	c.863 C>A	p.S288X	2.85	DC	-	0.00	Novel
KNUF29	*MYO1F*	14	Missense	c.1504 A>G	p.I502V	1.47	DC	Tolerated / 0.50	0.00	Zadro et al., 2009
KNUF34	*WFS1*	8	Missense	c.2209G>A	p.E737K	2.57	DC	Tolerated / 0.15	0.03^*^	Ohtsuki et al., 2000
KNUF46	*DIAPH1*	16	Missense	c.2032 C>T	p.P678S	1.12	DC	Affected / 0.00	0.00	Novel
KNUF57	*POU4F3*	2	Missense	c.694G>A	p.E232K	2.34	DC	Affected / 0.00	0.00	Novel
KNUF60	*COL11A2*	59	Missense	c.4265 C>T	p.P1422L	1.96	PO	Affected / 0.03	0.01^*^	Novel

* p.E737K in *WFS1* and p.P1422L in *COL11A2* found in normal controls were eliminated from the final candidate mutations.

The p.S288X variant in *EYA4* was not predicted by SIFT because it is a nonsense variant.

^†^DC, disease causing; PO, polymorphism. ^‡^ score ranges from 0, damaging to 1, neutral.

**Figure 2:**
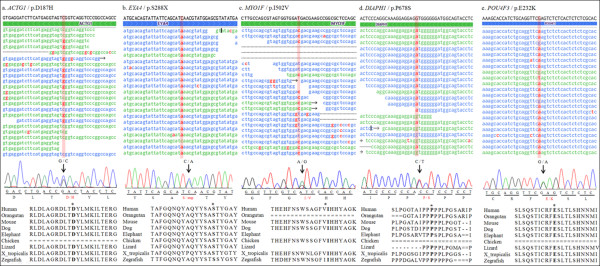
**The identification of 5 non-synonymous variations showing co-segregation with hearing loss in affected families.Top:** Visualization of individual sequencing reads covering the mutations in the genes. The actual read depth for these mutated nucleotides ranged between 46× and 442×. Blue and green reads represent the positive and negative strands, respectively, and the red bases represent bases that differ from the reference sequence (black). **Middle:** Verification of each variation by Sanger sequencing. All 5 of the variations marked by black arrows are single nucleotide substitutions leading to early truncation of the polypeptide or a change in the amino acid. **Bottom:** A comparison of amino acid sequences of each gene in multiple vertebrate species. The asterisks indicate the amino acid substituted by the mutation. A single line between the aligned amino acids indicates that there is no base in the aligned species, and a double line indicates that the aligned species has one or more bases that cannot be aligned in the gap region. The majority of the mutated amino acids are strongly conserved across the 9 different species shown.

To estimate the probable pathogenicity of the variants, evolutionary conservation and the predicted damaging effects of the amino acid substitution were analyzed. In most of the variants, the amino acid where the substitution occurred was highly conserved in a number of vertebrates showing positive PhyloP scores (Figure 2). In addition, two prediction programs, Mutation Taster and SIFT, strongly predicted that the variants would interrupt normal protein function (Table 3). However, one variant, proline at position 1422 in the *COL11A2* gene, was not conserved between other species and was predicted to be a probable polymorphism using Mutation Taster. Control sequencing of 100 unrelated normal individuals practically supported these predictions. However, we excluded two variants, the p.E737K variant in *WFS1* and the p.P1422L variant in *COL11A2* from the candidate mutations, because these two variants were detected in 5 and 2 normal controls, respectively (Table 3). In addition, p.E737K in *WFS1* was also detected in normal European American population (rs147834269) in the Washington University exome database ( http://evs.gs.wathington.edu). Furthermore, it has been reported to be a non-pathogenic polymorphism in several studies that provided strong evidence that this variant was not the genetic cause of hearing loss in the KNUF34 family [19,20]. In contrast, the other 5 variations (p.D187H in *ACTG1*, p.S288X in *EYA4*, p.I502V in *MYO1F*, p.P678S in *DIAPH1* and p.E232K in *POU4F3*) were absent in all 100 normal controls in this study. In addition, none of the healthy controls in the 1000 genomes project and the Washington University exome database carried these variants, suggesting that they could be pathogenic mutations.

The p.D187H variation in *ACTG1* was detected in patient III-1 of the KNUF21 family and followed an autosomal dominant inheritance pattern (Figure 2a). This variation was observed in 17 of 39 reads (44%) and had a quality score of 35, indicating heterozygosity. Genotype confirmation using Sanger sequencing revealed that two patients, II-2 and III-1, were heterozygous for this mutation. A normal individual, II-1, was homozygous for the wild type, consistent with the autosomal dominant inheritance pattern of the disorder.

The p.S288X mutation in *EYA4* was identified in the KNUF24 family (Figure 2b). In the targeted sequencing, the p.S288X mutation appeared in 45% of reads (21 of 47) and had a variation score of 35, indicating heterozygosity. Sanger sequencing revealed that this mutation is co-segregated with hearing loss in one patient (II-5) and was absent from two unaffected members (II-2 and III-2).

The KNUF29 family had dominant hearing loss that was confirmed to be caused by the p.I502V mutation in *MYO1F* (Figure 2c)*.* The heterozygosity and reliability of this mutation was supported by a 35% variation frequency (17 of 46) and a variation score of 35. This result was confirmed by Sanger sequencing, and only patient II-1 had the mutation, confirming its co-segregation with the disorder.

The KNUF46 family had low-to-mid-frequency non-syndromic hearing loss (Additional file 2: Figure S1). This type of hearing loss is uncommon and has been associated with only the *DIAPH1*, *MYO7A* and *WFS1* genes [21]. Interestingly, we identified the missense mutation, p.P678S of *DIAPH1* in this family (Figure 2d), and it showed coincident audiographical configuration with known DFNA1 (*DIAPH1*) hearing loss. This missense mutation was observed in 53 of 121 reads, with a variation score of 40. Site-directed sequencing confirmed that only the hearing loss patients in this family (of 4 patients and 2 controls sequenced) were heterozygous for the mutation.

A novel missense mutation, p.E232K in *POU4F3*, was identified in the KNUF57 family (Figure 2e). Thirty-six percent of all reads had this mutation, with a variation score of 35 in the targeted sequencing. Five family members, including 4 patients, were sequenced for the mutation by Sanger sequencing, which verified co-segregation.

In summary, the targeted sequencing of 15 individuals from 8 DFNA families yielded 10 candidate non-synonymous mutations as the primary causes of hearing loss. Sanger sequencing was carried out in 31 individuals, including 16 additional family members, to confirm whether these 10 variations are co-segregated with hearing loss. This sequencing allowed us to confirm the 7 co-inherited variations. Finally, variation screening in 100 normal controls using Sanger sequencing confirmed 5 variations as probable pathogenic mutations in 5 of the families.

## Discussion

The purpose of this study was to identify the genetic basis of hereditary hearing loss using next-generation sequencing technology in small families who could not be analyzed using the current genetic approaches. Eight small families with autosomal dominant, non-syndromic, sensorineural hearing loss were selected, and 80 target genes associated with hearing loss were screened using target capture and massively parallel sequencing methods. In current study, 5 non-synonymous mutations were confirmed in 5 of the 8 families. The causative genes underlying the hearing loss in the 3 other families are still awaiting discovery. There are 2 possible explanations for the hearing loss in these families: (1) pathogenic mutations exist in 1 of the 80 candidate genes studied but in an exon that was not covered by our sequencing (approximately 6-10%) or within intronic regulatory sequences, (2) their causative mutation is in an as-yet-unidentified hearing loss gene. However, 5 distinct causative mutations were identified in the other 5 families, including 4 missense mutations and 1 nonsense mutation. None of these mutations, except the p.I502V mutation in *MYO1F*, has been identified in previous studies of hereditary hearing loss.

*ACTG1* (NM_001199954)*,* which is responsible for DFNA20/26 hearing loss, encodes γ-actin, one of the non-muscle cytoskeletal proteins. This protein is predominantly expressed in cochlear hair cells and contributes to the structural maintenance of stereocilia, cuticular plates and adherens junctions [22]. γ-actin consists of 4 sub-domains (sub-domains 1-4), and the novel missense mutation p.D187H that is caused by a c.559 G > C transversion results in the substitution of a basic amino acid (histidine) for an acidic amino acid (aspartic acid) in sub-domain 4. Otterbein et al. found that even minor changes in this domain may lead to major effects on the structural stability of the actin polymer [23]. Using 2D gel electrophoresis, Verrills et al. identified that p.D187H in *ACTG1* leads to the expression of more basic gamma-actin in leukemia cells, and they reported that Asp187 is a surface residue in close proximity to the ATP-binding cleft of the protein. The substitution of the histidine for aspartic acid in *ACTG1* changes the charge from negative to positive, which results in reduced hydrophobicity and electrostatic interactions in this region. According to their research, mutant gamma-actin expressing leukemia cells display resistance to anti-microtubule drugs. The mutant gamma-actin expressing cells intactly retained their morphology, whereas the cells expressing wild-type gamma-actin were contracted and destroyed. These data suggest that the Asp187 residue in gamma-actin contributes to the interaction with microtubules, and the p.D187H variant inhibits depolymerization of tubulin in leukemia cells. This result suggests that the p.D187H variant could collapse the polymerization-depolymerization balance of microtubules, which leads to the destruction of cellular homeostasis in normal hair cells [24]. These conclusions from previous biochemical studies provide convincing evidence that p.D187H is a novel mutation that has pathogenic effects on the normal functions of γ-actin in the hair cells.

Mutation p.S288X in *EYA4* (NM_004100.4) was the only nonsense mutation identified in this study. The protein encoded by *EYA4* (DFNA10) is a member of the vertebrate Eya family of transcriptional activators, and it consists of two functional domains: the C-terminal EYA homolog domain and the N-terminal transactivation domain [13]. The EYA homolog domain and SIX family transcription factors interact to form transcriptional complexes that regulate the expression of target genes that are required for the development and maturation of the organ of Corti [13]. Most of the reported *EYA4* mutations produce truncated proteins missing a part of the EYA homolog domain [25]. The novel nonsense mutation p.S288X changed Ser288 to a stop codon, which produced a truncated protein lacking the entire EYA homolog domain. It suggests that this nonsense mutation may inhibit normal development and maintenance of the organ of Corti and cause sensorineural hearing loss.

*MYO1F* (NM_012335.3) has been frequently proposed as a candidate hearing loss gene, because several myosin genes have been demonstrated as causative genes of non-syndromic hearing loss and *MYO1F* is expressed in cochlea. Recently, Zadro et al. reported that *MYO1F* mutations were identified in hearing loss patients, and one of the reported mutations, p.I502V, was detected in this study [14]. According to their study, the mutated residue, Ile502, is located near the actin-binding site in the motor domain of myosin-1 f. Through homology modeling, it was predicted that the Ile502 residue contributes to the structural stabilization of the protein by forming hydrophobic interactions with the Val444, Leu447 and Ile448 residues. Additionally, there is an ATP-binding site near Ile502. Therefore, the authors concluded that the substitution of isoleucine for valine may interrupt the hydrophobic interaction with other residues, resulting in structural instability of the protein and disturbance of ATP binding. Based on these predictions, it can be hypothesized that the p.I502V mutation likely has a pathogenic effect on the cellular function of myosin-1 F [14].

Human diaphanous 1 protein, encoded by *DIAPH1* (NM_005219.4), belongs to the formin protein family, which regulates various cellular mechanisms such as cytoskeleton remodeling and the maintenance of cell polarity in hair cells [16,26-29]. The formins have several functional domains, including formin homology-1 and 2 domains (FH1, FH2), which play a key role in the polymerization of unbranched actin filaments by interacting with profilin [26,30,31]. The FH1 domain is characterized by consecutive proline residues. Profilin-actin complexes bind to the poly-L-proline stretch of the FH1 domain and are assembled into unbranched actin filaments allowing barbed end elongation [32]. The novel mutation p.P678S is located in the poly-L-proline stretch of the FH1 domain. This mutation changes a hydrophobic non-polar residue (proline) to a hydrophilic polar residue (serine) in the poly-L-proline stretch, and it will likely adversely affect actin polymerization at the barbed end. Therefore, it can be hypothesized that this mutation may disturb the interactions with partner proteins, which interrupts actin polymerization and collapses cellular polarity in hair cells of cochlea.

Transcription factors bind directly to DNA and regulate expression of target genes. Brn-3.1, encoded by the *POU4F3* gene (NM_002700.2), is a well-known transcription factor that contributes to the differentiation and survival of hair cells in cochlea [33,34]. This protein is composed of two functional DNA-binding domains: a POU-specific domain and a POU-homeodomain. In the DNA binding domains, the molecular features of amino acids such as electric property and acidity significantly influence the maintenance of the structural conformation of the protein [34,35]. The p.E232K mutation detected in this study is caused by a single nucleotide substitution of adenine for guanine at nucleotide position 694, resulting in the replacement of a negatively charged glutamic acid with a positively charged lysine in an α-helix (α3) in the POU-specific domain. According to 3D modeling by Collin et al., among the 4 α-helix structures in the POU-specific domain, the α3 helix where the variant p.E232K occurs has the most direct interaction with target DNA [34]. Thus, altered electric properties of the Glu232 residue would directly reduce the structural stability of the domain or the strength of the interaction with the target DNA. It suggests that this missense mutation might have pathogenic effects on the maturation and survival of hair cells by failing to regulate the expression of downstream genes.

Although the pedigrees have insufficient genetic information, various probable pathogenic mutations were successfully detected by NGS technique and molecular genetic analysis in current study. Moreover, it will completely overcome the weakness of this study ensuring pathogenicity of the mutations when segregation study for the detected mutations can be performed with all of the members in the family.

According to a number of previous genetic studies, hereditary hearing loss shows distinct spectrums and prevalence of mutations in different ethnic groups [36-40]. For example, the *GJB2* gene accounts for approximately 20-40% of genetic hearing loss in Caucasian populations but only 10% of genetic hearing loss in Korean populations [37,38,41-43]. In addition, the most frequent *GJB2* mutation also varies among populations: c.35delG, c.235delC and c.167delT are the most common variants in Caucasian, Asian and Jewish populations, respectively [38,44-46]. However, this feature may not be applicable for autosomal dominant hearing loss because major hearing loss genes such as *GJB2* and *SLC26A4* predominantly cause autosomal recessive hearing loss. To date, no major genes have been described that cause a significant proportion of dominant hearing loss in any ethnic population. In this study, 5 mutations were detected in 5 different genes in 5 Korean families with autosomal dominant, non-syndromic, sensorineural hearing loss. Additionally, we provide the first evidence of pathogenic mutations in the *ACTG1, EYA4, DIAPH1* and *MYO1F* genes in a Korean population. These results, as well as previous genetic studies performed by our group, suggest that there are no mutational hot spots for dominant hearing loss in the Korean population [33,47-50]. This conclusion is consistent with the idea that the genetic causes of autosomal dominant hearing loss are more heterogeneous than those of autosomal recessive hearing loss in most ethnic groups.

Because of these characteristics, it is difficult to diagnose and establish the exact causes of hereditary hearing loss, although it accounts for approximately 50% of all hearing loss [51]. Currently, several simple DNA tests are performed in medical institutions in many countries to diagnose hereditary hearing loss. However, the test evaluates only few major genes, such as *GJB2, SLC26A4* and mitochondrial genes, and is unable to detect other genetic causes of hereditary hearing loss. Considering that the ultimate goal of genetic disease research is to establish basic information and genetic databases for clinical diagnosis and treatment, the use of population genetic studies to accurately understand the genetic background of diseases is essential. Although current genetic technologies, such as linkage analysis and Sanger sequencing, are very reliable methods for identifying genomic variations associated with genetic disorders, they are not well suited for the analysis of heterogeneous diseases. Next-generation sequencing (NGS) is accelerating the qualitative improvement of mutational studies for numerous heterogeneous disorders due to its ability to perform simultaneous and massively parallel sequencing. Although the current NGS technique is too expensive to be widely used, the cost of NGS is gradually decreasing, which will lead to the increased applicability of this technology. Two recent population genetic studies on hereditary hearing loss have been performed in American and Jewish populations [52,53]. In these studies, various genetic mutations and a founder mutation were detected using next-generation sequencing. Our study is the first report of dominant hearing loss causative gene mutations identified by the targeted-sequencing of affected individuals in an East Asian population. Our successful identification of several pathogenic mutations using target-capture and massively parallel sequencing demonstrates that gene targeted sequencing is a highly effective and powerful tool for clinical and population genetic studies of heterogeneous disorders.

## Competing interests

The authors declare that they have no competing interests.

## Authors’ contributions

JI participated in the selection of target genes, performed bioinformatic interpretation of the NGS data, confirmed the NGS data through Sanger sequencing and has drafted the manuscript. SK interpreted the NGS data and confirmed the NGS data by Sanger sequencing. DB extracted genomic DNA of all participants from their whole blood and performed bioinformatic interpretation of the NGS data. SY participated in the selection of target genes and predicted probable pathogenic effects of the mutations by several software programs. UK has made general study design and wrote the manuscript. KY has directed all clinical parts including clinical evaluation and hearing test, has made study design. SH clinically investigated the patients and collected genetic information of all families participated in this study. All authors read and approved the final manuscript.

## Supplementary Material

Additional file 1 **Table S1.** Eighty genes targeted for the next-generation sequencing. Click here for file

Additional file 2 **Figure S1.** Audiograms for PTA (pure-tone audiometry) thresholds of 7 probands (A – G) and a normal family member (H. KNUF24: III-2). The most of probands except II-3 of KNUF60 (G), show symmetrical bilateral high frequency hearing loss, which is general audiographical aspect of autosomal dominant non-syndromic hearing loss. The proband III-1 of KNUF46 who shows low-to-mid frequency hearing loss, carries a missense mutation (p.P678S) in *DIAPH1* gene which causes low frequency hearing loss. It provides a strong possibility that p.P678S is the pathogenic mutation causing hereditary hearing loss in family KNUF46. Click here for file

Additional file 3 **Table S2.** Summary of genetic variations detected in targeted sequencing. Click here for file
