# Supplementary material for: Targeted massive parallel sequencing: the effective detection of novel causative mutations associated with hearing loss in small families
Source: Orphanet J Rare Dis. 2012 Sep 3;7:60. doi: 10.1186/1750-1172-7-60 (PMC3495859; doi:10.1186/1750-1172-7-60)
Supplement: Additional file 1 — Table S1. Eighty genes targeted for the next-generation sequencing. [file 1750-1172-7-60-S1.doc]

**Table S1.** Eighty genes targeted for the next-generation sequencing.

| **Hearing loss**  **locus*** | **Chromosomal**  **position** | **Gene symbol** | **mRNA length (bp)** | **Reference** |
| --- | --- | --- | --- | --- |
| A1 | 5q31 | *DIAPH1* | 5662 | Lynch et al., 1997 [1] |
| A2A | 1p34 | *KCNQ4* | 2335 | Kubisch et al., 1999 [2] |
| A2B | 1p35.1 | *GJB3* | 1777 | Xia et al., 1998 [3] |
| A3A/B1A | 13q11-q12 | *GJB2* | 2263 | Zelante et al., 1997 [4] |
| A3B/B1B | 13q12 | *GJB6* | 1805 | Grifa et al., 1999; Castillo et al., 2002 [5, 6] |
| A4 | 19q13 | *MYH14* | 6786 | Donaudy et al., 2004 [7] |
| A5 | 7p15 | *DNFA5* | 2230 | Van laer et al., 1998 [8] |
| A6/A14/A38 | 4p16.3 | *WFS1* | 3640 | Bespalova et al., 2001 [9] |
| A8/A12/B21 | 11q22-q24 | *TECTA* | 6469 | Verhoeven et al., 1998; Mustapha et al., 1999 [10, 11] |
| A9 | 14q12-q13 | *COCH* | 2534 | Robertson et al., 1998 [12] |
| A10 | 6q22-q23 | *EYA4* | 3077 | Wayne et al., 2001 [13] |
| A11/B2 | 11q12.3-q21 | *MYO7A* | 7465 | Liu et al., 1997; Weil et al., 1997 [14] |
| A13/B53 | 6p21 | *COL11A2* | 6414 | McGuirt et al., 1999; Chen et al., 2005 [15, 16] |
| A15 | 5q31 | *POU4F3* | 1017 | Vahava et al., 1998 [17] |
| A17 | 22q | *MYH9* | 7474 | Lalwani et al., 2000 [18] |
| A20/A26 | 17q25 | *ACTG1* | 1919 | Zhu et al., 2003 [19] |
| A22/B37 | 6q13 | *MYO6* | 5278 | Melchionda et al., 2001; Ahmed et al., 2003 [20, 21] |
| A28 | 8q22 | *GRHL2* | 5231 | Peters et al., 2002 [22] |
| A36/B7/B11 | 9q13-q21 | *TMC1* | 3201 | Kurima et al., 2002; Jain et al., 1995 [23, 24] |
| A39 | 4q21.3 | *DSPP* | 4187 | Xiao et al., 2001 [25] |
| A44 | 3q28-q29 | *CCDC50* | 2454 | Modamio-Hoybjor et al., 2007 [26] |
| A48 | 12q13-q14 | *MYO1A* | 3624 | Donaudy et al., 2003 [27] |
| B3 | 17p11.2 | *MYO15A* | 11876 | Liang et al., 1999 [28] |
| B4 | 7q31 | *SLC26A4* | 4930 | Li et al., 1998 [29] |
| B6 | 3p14-p21 | *TMIE* | 1646 | Naz et al., 2002 [30] |
| B8/B10 | 21q22 | *TMPRSS3* | 2468 | Scott et al., 2001 [31] |
| B9 | 2p22-p23 | *OTOF* | 7173 | Yasunaga et al., 1999 [32] |
| B12 | 10q21-q22 | *CDH23* | 11073 | Bork et al., 2001 [33] |
| B16 | 15q21-q22 | *STRC* | 5516 | Verpy et al., 2001 [34] |
| B18 | 11p14-p15.1 | *USH1C* | 2228 | Ouyang et al., 2002; Ahmed et al., 2002 [35, 36] |
| B22 | 16p12.2 | *OTOA* | 3625 | Zwaenepoel et al ., 2002 [37] |
| B23 | 10p11.2-q21 | *PCDH15* | 7022 | Ahmed et al, 2003 [38] |
| B24 | 11q23 | *RDX* | 4498 | Khan et al., 2007 [39] |
| B28 | 22q13 | *TRIOBP* | 10024 | Shahin et al., 2006 [40] |
| B29 | 21q22 | *CLDN14* | 1943 | Wilcox et al., 2001 [41] |
| B30 | 10p11.1 | *MYO3A* | 5597 | Walsh et al., 2002 [42] |
| B31 | 9q32-q34 | *WHRN* | 4022 | Mburu et al., 2003 [43] |
| B35 | 14q24.1-q24.3 | *ESRRB* | 2193 | Collin et al., 2008 [44] |
| B36 | 1p36.3 | *ESPN* | 3542 | Naz et al., 2004 [45] |
| B49 | 5q12.3-q14.1 | *MARVELD2* | 2385 | Riazuddin et al., 2006 [46] |
| B59 | 2q31.1-q31.3 | *PJVK* | 1415 | Delmaghani et al., 2006 [47] |
| B61 | 7q22.1 | *SLC26A5* | 2671 | Liu et al., 2003 [48] |
| B63 | 11q13.2-q13.4 | *LRTOMT* | 2332 | Ahmed et al., 2008 [49] |
| B66/B67 | 6p21.2-p22.3 | *LHFPL5* | 2162 | Kalay et al., 2006 [50] |
| B73 | 1p32.3 | *BSND* | 1396 | Riazuddin et al., 2009 [51] |

Continued

| **Hearing loss**  **locus*** | **Chromosomal**  **position** | **Gene symbol** | **mRNA length (bp)** | **Reference** |
| --- | --- | --- | --- | --- |
| - | 7p22 | *ACTB* | 1852 | Perrin et al., 2010 [52] |
| - | 2p13.1 | *ATP6V1B1* | 1956 | Stover et al., 2002 [53] |
| - | 2q33 | *BCS1L* | 1663 | Hinson et al., 2007 [54] |
| - | 15q15.3 | *CATSPER2* | 1948 | Zhang et al., 2007 [55] |
| - | 20q13.3 | *COL9A3* | 2485 | Asamura et al., 2005 [56] |
|  | 16p | *CRYM* | 1303 | Abe et al., 2003 [57] |
| - | 19q13.3 | *ERCC2* | 2568 | Flores-Alvarado et al., 2010 [58] |
| - | 2q21 | *ERCC3* | 2751 | Flores-Alvarado et al., 2010 [58] |
| - | 11q13 | *FGF3* | 1548 | Alsmadi et al., 2009 [59] |
| - | 10p15 | *GATA3* | 3067 | Ferraris et al., 2009 [60] |
| - | 6q21-q23.2 | *GJA1* | 3130 | Liu et al., 2001 [61] |
| - | Xq22 | *GJB1* | 1623 | Matsuyama et al., 2001 [62] |
| - | 1p34.3 | *GJB4* | 2840 | Lopez-Bigas et al., 2002 [63] |
| - | 11q13 | *GSTP1* | 986 | Ates et al., 2005 [64] |
| - | 20p12-p11.23 | *JAG1* | 5988 | Kiernan et al., 2006 [65] |
| JLNS2 | 21q22.12 | *KCNE1* | 3338 | Knipper et al., 2006 [66] |
| - | 1q23.1 | *KCNJ10* | 5323 | Reichold et al., 2010 [67] |
|  | 9p34.4 | *LHX3* | 2376 | Rajab et al., 2008 [68] |
| - | 9p21 | *MTAP* | 4937 | Williamson et al., 2007 [69] |
| - | 17p13.3 | *MYO1C* | 4973 | Zadro et al., 2009 [70] |
| - | 19p13.3.-p13.2 | *MYO1F* | 4173 | Zadro et al., 2009 [70] |
| - | 5q14 | *NR2F1* | 3210 | Brown et al., 2009 [71] |
| - | 20p12.1-p11.23 | *OTOR* | 1477 | Rendtorff et al., 2001 [72] |
| WS1 | 2q35 | *PAX3* | 3359 | Baldwin et al., 1995 [73] |
|  | 10q24.31 | *PDZD7* | 2072 | Ebermann et al., 2010 [74] |
| - | 17p12 | *PMP22* | 1828 | Verhagen et al., 2005 [75] |
| X2 | Xq21.1 | *POU3F4* | 1491 | de Kok et al., 1995 [76] |
| - | 12q23.1 | *SLC17A8* | 3983 | Ruel et al., 2008 [77] |
| - | 20p12 | *SLC4A11* | 3110 | Lopez et al., 2009 [78] |
| - | 3q26.3-q27 | *SOX2* | 2518 | Puligilla et al., 2010 [79] |
| - | 5q32 | *SPINK5* | 3655 | Chavanas et al., 2000 [80] |
| - | Xp22.3 | *TBL1X* | 5586 | Yan et al., 2005 [81] |
| - | 6q23-q24 | *TCF21* | 3249 | Arbustini et al., 2005 [82] |
| - | Xq22.1 | *TIMM8A* | 1492 | Tranebjaerg et al., 2001 [83] |
| - | 11q | *TMPRSS5* | 2233 | Guipponi et al., 2008 [84] |

*A, DFNA (dominant hearing loss) locus; B, DFNB (recessive hearing loss) locus; WS, Waardenburg syndrome; JLNS, Jervell & Lange-Nielsen syndrome; X, DFNX (X-linked hearing loss) locus

.
