# Supplementary material for: Targeted massive parallel sequencing: the effective detection of novel causative mutations associated with hearing loss in small families
Source: Orphanet J Rare Dis. 2012 Sep 3;7:60. doi: 10.1186/1750-1172-7-60 (PMC3495859; doi:10.1186/1750-1172-7-60)
Supplement: Additional file 2 — Figure S1. Audiograms for PTA (pure-tone audiometry) thresholds of 7 probands (A – G) and a normal family member (H. KNUF24: III-2). The most of probands except II-3 of KNUF60 (G), show symmetrical bilateral high frequency hearing loss, which is general audiographical aspect of autosomal dominant non-syndromic hearing loss. The proband III-1 of KNUF46 who shows low-to-mid frequency hearing loss, carries a missense mutation (p.P678S) in DIAPH1 gene which causes low frequency hearing loss. It provides a strong possibility that p.P678S is the pathogenic mutation causing hereditary hearing loss in family KNUF46. [file 1750-1172-7-60-S2.doc]

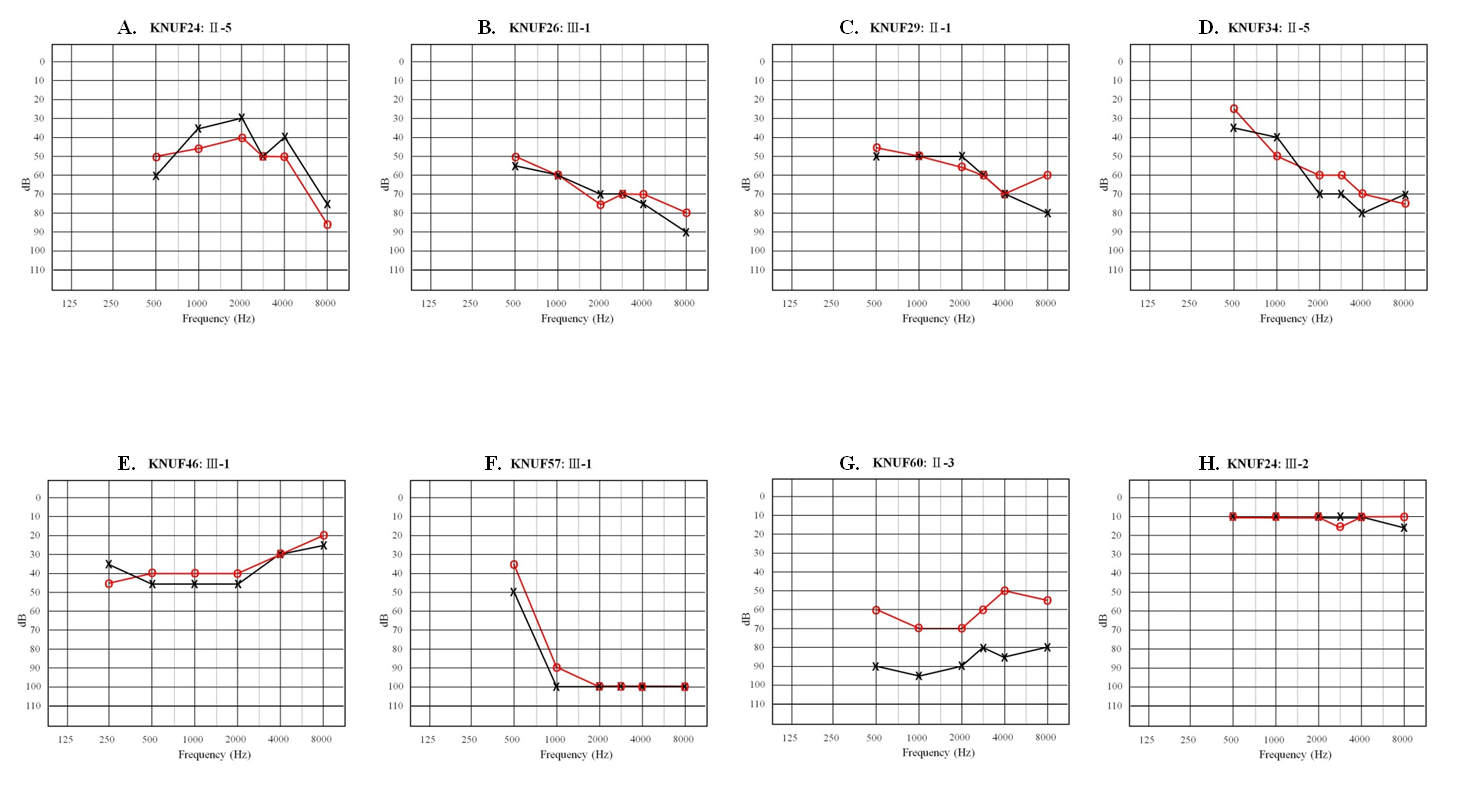


**Figure S1**. Audiograms for PTA (pure-tone audiometry) thresholds of 7 probands (A – G) and a normal family member (H. KNUF24: III-2). The most of probands except II-3 of KNUF60 (G), show symmetrical bilateral high frequency hearing loss, which is general audiographical aspect of autosomal dominant non-syndromic hearing loss. The proband III-1 of KNUF46 who shows low-to-mid frequency hearing loss, carries a missense mutation (p.P678S) in *DIAPH1* gene which causes low frequency hearing loss. It provides a strong possibility that p.P678S is the pathogenic mutation causing hereditary hearing loss in family KNUF46.
