# Supplementary material for: Targeted massive parallel sequencing: the effective detection of novel causative mutations associated with hearing loss in small families
Source: Orphanet J Rare Dis. 2012 Sep 3;7:60. doi: 10.1186/1750-1172-7-60 (PMC3495859; doi:10.1186/1750-1172-7-60)
Supplement: Additional file 3 — Table S2. Summary of genetic variations detected in targeted sequencing. [file 1750-1172-7-60-S3.doc]

**Table S2.** Summary of genetic variations detected in targeted sequencing.

| Family no. | Individual no. | Total variations | |  | Novel | |  | | CDS | | | | |
| --- | --- | --- | --- | --- | --- | --- | --- | --- | --- | --- | --- | --- | --- |
|  |  | dbSNP | **Novel** |  | Non-coding | **CDS** ^a^ | |  | | Syn.^b^ | **Ins/del** ^c^ | **Nonsense** | **Missense** |
| KNUF21 | Ⅲ-1 | 335 | 91 |  | 73 | 18 | |  | | 8 | **-** | **1** | **9** |
| KNUF24 | Ⅱ-2 | 475 | 97 |  | 84 | 13 | |  | | 2 | **1** | **-** | **10** |
|  | Ⅱ-5 | 397 | 117 |  | 83 | 34 | |  | | 11 | **-** | **3** | **20** |
| KNUF26 | Ⅱ-3 | 331 | 100 |  | 84 | 16 | |  | | 8 | **1** | **-** | **7** |
|  | Ⅲ-1 | 319 | 82 |  | 54 | 28 | |  | | 7 | **1** | **1** | **19** |
| KNUF29 | Ⅱ-1 | 294 | 95 |  | 79 | 16 | |  | | 4 | **-** | **1** | **11** |
|  | Ⅲ-1 | 490 | 124 |  | 95 | 29 | |  | | 14 | **1** | **-** | **14** |
| KNUF34 | Ⅱ-5 | 450 | 99 |  | 79 | 20 | |  | | 13 | **-** | **1** | **6** |
|  | Ⅲ-3 | 340 | 216 |  | 193 | 23 | |  | | 6 | **-** | **1** | **16** |
| KNUF46 | Ⅱ-2 | 344 | 69 |  | 55 | 14 | |  | | 7 | **1** | **1** | **5** |
|  | Ⅲ-1 | 266 | 78 |  | 61 | 17 | |  | | 5 | **1** | **-** | **11** |
| KNUF57 | Ⅱ-2 | 406 | 80 |  | 65 | 15 | |  | | 9 | **-** | **1** | **5** |
|  | Ⅲ-1 | 384 | 112 |  | 81 | 31 | |  | | 5 | **-** | **5** | **21** |
| KNUF60 | Ⅰ-1 | 184 | 101 |  | 85 | 16 | |  | | 8 | **-** | **-** | **8** |
|  | Ⅱ-3 | 402 | 299 |  | 276 | 23 | |  | | 10 | **-** | **-** | **13** |

^a^ CDS, coding sequence; ^b^ Syn, synonymous; ^c^ Ins / del, insertion or deletion.
